# 3-(2-Bromo­eth­yl)-5,5-di­phenyl­imidazolidine-2,4-dione

**DOI:** 10.1107/S2414314623000603

**Published:** 2023-01-26

**Authors:** Walid Guerrab, Abderrazzak El Moutaouakil Ala Allah, Abdulsalam Alsubari, Joel T. Mague, Youssef Ramli

**Affiliations:** a Laboratory of Medicinal Chemistry, Drug Sciences Research Center, Faculty of Medicine and Pharmacy, Mohammed V University in Rabat, Morocco; b Laboratory of Medicinal Chemistry, Faculty of Clinical Pharmacy, 21 September University, Yemen; c Department of Chemistry, Tulane University, New Orleans, LA 70118, USA; Sunway University, Malaysia

**Keywords:** crystal structure, imidazolidenedione, hydrogen bond, C—H⋯π(ring) inter­action

## Abstract

The imidazolidine ring in the title mol­ecule, C_17_H_15_BrN_2_O_2_, is slightly ruffled [r.m.s. deviation = 0.0192 Å], while the attached phenyl rings at the C atom at the position between the amine and carbonyl centres are rotated well out of its mean plane [dihedral angles with the imidazolidine ring = 63.60 (8) and 76.4 (1)°]. In the crystal, a three-dimensional network features N—H⋯O and C—H⋯O hydrogen bonds together with C—H⋯π(ring) inter­actions.

## Abstract 2

The imidazolidine ring is slightly ruffled while the attached phenyl rings are rotated well out of its mean plane. In the crystal, N—H⋯O hydrogen bonds form inversion dimers, which are connected into layers parallel to (101) by C—H⋯O hydrogen bonds. The layers are connected into a three-dimensional network by additional C—H⋯O hydrogen bonds and C—H⋯π(ring) inter­actions.

## Graphical abstract

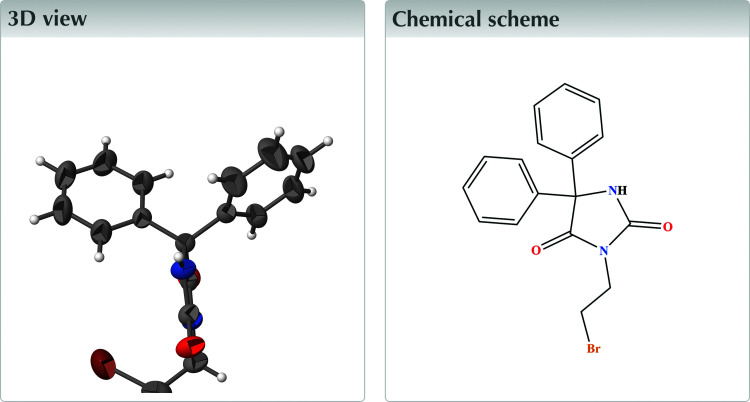

## Structure description

**Figure 1:**
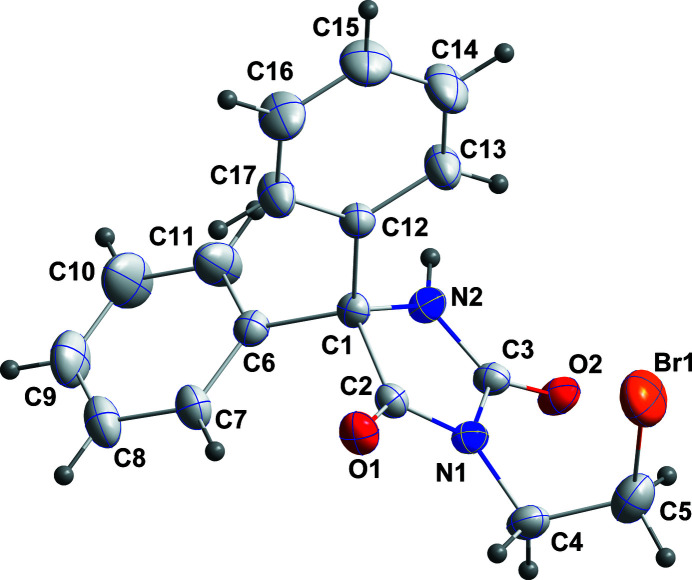
The title mol­ecule showing the atom-labelling scheme and 30% probability ellipsoids.

Phenytoine (5,5-di­phenyl­imidazolidine-2,4-dione) is a drug widely prescribed as an anti­convulsant agent and for the treatment of many other diseases, including HIV (Weichet, 1974 ▸; Havera & Strycker, 1976 ▸; Khodair *et al.*, 1997 ▸; Thenmozhiyal *et al.*, 2004 ▸). Given the wide range of therapeutic applications for such compounds, and in a continuation of our work in this area (Ramli *et al.*, 2017*a* ▸,*b* ▸; Akrad *et al.*, 2017 ▸; Guerrab *et al.*, 2019 ▸, 2020*a* ▸,*b* ▸, 2022*a* ▸,*b* ▸), the title compound (Fig. 1 ▸) was prepared and its crystal structure determined.

**Figure 2:**
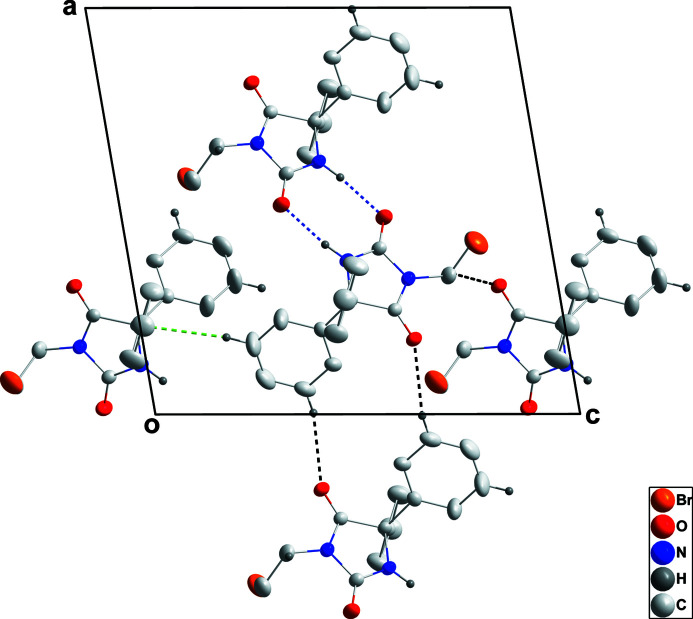
Detail of the inter­molecular inter­actions viewed along the *b*-axis direction. N—H⋯O and C—H⋯O hydrogen bonds are shown, respectively, by blue and black dashed lines, while the C—H⋯π(ring) inter­actions are shown by green dashed lines.

**Figure 3:**
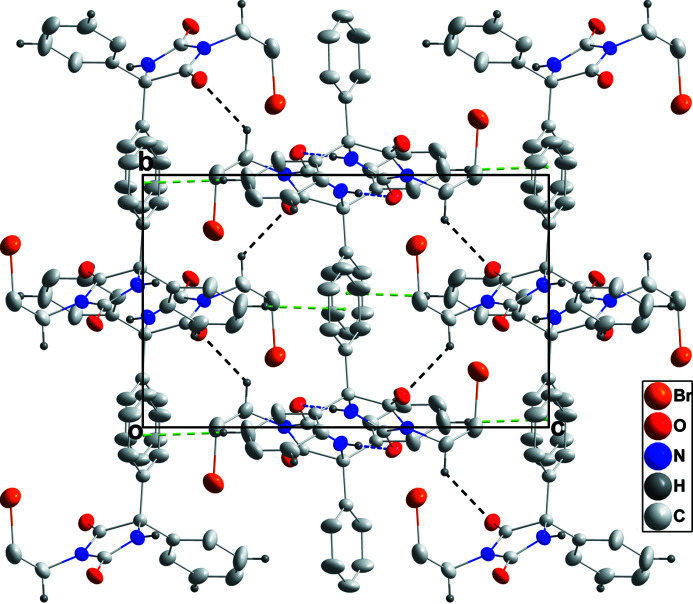
Packing viewed along the *a-*axis direction with inter­molecular inter­actions depicted as in Fig. 2 ▸.

The C1/C2/N1/C3/N2 ring is planar to within 0.0254 (13) Å (r.m.s. deviation of the fitted atoms = 0.0192 Å) with the atoms alternately disposed above and below the mean plane. The C6–C11 and C12–C17 phenyl rings are inclined at 63.60 (8) and 76.4 (1)°, respectively, to the the above plane. In the crystal, inversion dimers are formed by N2—H2⋯O2 hydrogen bonds (Table 1 ▸ and Fig. 2 ▸) and are connected into layers parallel to (101) by C4—H4*A*⋯O1 hydrogen bonds (Table 1 ▸ and Fig. 2 ▸). These layers are joined into a three-dimensional network by C8—H8⋯O1 hydrogen bonds and C10—H10⋯*Cg*(C12–C17) inter­actions (Table 1 ▸ and Fig. 3 ▸).

## Synthesis and crystallization

To a solution of 5,5-di­phenyl­imidazolidine-2,4-dione (500 mg, 1.98 mmol), one equivalent of 1,2-di­bromo­ethane (171.58 ml, 1.98 mmol), in absolute di­methyl­formamide (DMF, 15 ml), was added and the resulting solution heated under reflux for 3 h in the presence of 1.2 equivalents of K_2_CO_3_ (331.20 mg, 2.37 mmol). The reaction mixture was filtered while hot, and the solvent evaporated under reduced pressure. The residue obtained was dried and recrystallized from an ethanol solution to yield colourless blocks (Guerrab *et al.*, 2018 ▸).

## Refinement

Crystal and refinement details are presented in Table 2 ▸.

## Supplementary Material

Crystal structure: contains datablock(s) global, I. DOI: 10.1107/S2414314623000603/tk4088sup1.cif

Structure factors: contains datablock(s) I. DOI: 10.1107/S2414314623000603/tk4088Isup2.hkl

Supporting information file. DOI: 10.1107/S2414314623000603/tk4088Isup3.cml

CCDC reference: 2235944

Additional supporting information:  crystallographic information; 3D view; checkCIF report

## Figures and Tables

**Table 1:** Hydrogen-bond geometry (Å, °) *Cg*3 is the centroid of the C12–C17 benzene ring.

*D*—H⋯*A*	*D*—H	H⋯*A*	*D*⋯*A*	*D*—H⋯*A*
N2—H2⋯O2^i^	0.89	1.98	2.862 (3)	174
C4—H4*A*⋯O1^ii^	0.97	2.49	3.175 (3)	128
C8—H8⋯O1^iii^	0.93	2.56	3.387 (3)	148
C10—H10⋯*Cg*3^iv^	0.93	2.85	3.771 (5)	173

Symmetry codes: (i) 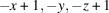 ; (ii) 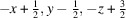 ; (iii) 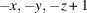 ; (iv) 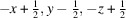 .

**Table 2:** Experimental details

Crystal data	
Chemical formula	C_17_H_15_BrN_2_O_2_
*M*_r_	359.22
Crystal system, space group	Monoclinic, *P*2_1_/*n*
Temperature (K)	298
*a*, *b*, *c* (Å)	13.7083 (5), 8.6500 (3), 14.1183 (5)
β (°)	99.724 (1)
*V* (Å^3^)	1650.05 (10)
*Z*	4
Radiation type	Mo *K*α
μ (mm^−1^)	2.50
Crystal size (mm)	0.42 × 0.32 × 0.25

Data collection	
Diffractometer	Bruker SMART APEX CCD
Absorption correction	Multi-scan (*SADABS*; Krause *et al.*, 2015 ▸)
*T*_min_, *T*_max_	0.46, 0.58
No. of measured, independent and observed [*I* > 2σ(*I*)] reflections	30580, 4284, 3056
*R*_int_	0.028
(sin θ/λ)_max_ (Å^−1^)	0.678

Refinement	
*R*[*F* ^2^ > 2σ(*F* ^2^)], *wR*(*F* ^2^), *S*	0.052, 0.170, 1.06
No. of reflections	4284
No. of parameters	199
H-atom treatment	H-atom parameters constrained
Δρ_max_, Δρ_min_ (e Å^−3^)	0.82, −0.67

Computer programs: *APEX3* and *SAINT* (Bruker, 2016 ▸), *SHELXT2014/5* (Sheldrick, 2015*a* ▸), *SHELXL2018/1* (Sheldrick, 2015*b* ▸) and *DIAMOND* (Brandenburg & Putz, 2012 ▸).

## full crystallographic data

### Crystal data

**Table:**

C_17_H_15_BrN_2_O_2_	*F*(000) = 728
*M**_r_* = 359.22	*D*_x_ = 1.446 Mg m^−^^3^
Monoclinic, *P*2_1_/*n*	Mo *K*α radiation, λ = 0.71073 Å
*a* = 13.7083 (5) Å	Cell parameters from 9961 reflections
*b* = 8.6500 (3) Å	θ = 2.3–27.3°
*c* = 14.1183 (5) Å	µ = 2.50 mm^−^^1^
β = 99.724 (1)°	*T* = 298 K
*V* = 1650.05 (10) Å^3^	Block, colourless
*Z* = 4	0.42 × 0.32 × 0.25 mm

### Data collection

**Table:**

Bruker SMART APEX CCD diffractometer	4284 independent reflections
Radiation source: fine-focus sealed tube	3056 reflections with *I* > 2σ(*I*)
Graphite monochromator	*R*_int_ = 0.028
Detector resolution: 8.3333 pixels mm^-1^	θ_max_ = 28.8°, θ_min_ = 1.9°
φ and ω scans	*h* = −18→17
Absorption correction: multi-scan (*SADABS*; Krause *et al.*, 2015)	*k* = −11→11
*T*_min_ = 0.46, *T*_max_ = 0.58	*l* = −19→19
30580 measured reflections	

### Refinement

**Table:**

Refinement on *F*^2^	Primary atom site location: structure-invariant direct methods
Least-squares matrix: full	Secondary atom site location: difference Fourier map
*R*[*F*^2^ > 2σ(*F*^2^)] = 0.052	Hydrogen site location: mixed
*wR*(*F*^2^) = 0.170	H-atom parameters constrained
*S* = 1.06	*w* = 1/[σ^2^(*F*_o_^2^) + (0.0893*P*)^2^ + 0.7858*P*] where *P* = (*F*_o_^2^ + 2*F*_c_^2^)/3
4284 reflections	(Δ/σ)_max_ = 0.001
199 parameters	Δρ_max_ = 0.82 e Å^−^^3^
0 restraints	Δρ_min_ = −0.67 e Å^−^^3^

### Special details

**Table:**

Experimental. The diffraction data were obtained from 3 sets of 400 frames, each of width 0.5° in ω, colllected at φ = 0.00, 90.00 and 180.00° and 2 sets of 800 frames, each of width 0.45° in φ, collected at ω = –30.00 and 210.00°. The scan time was 15 sec/frame.
Geometry. All esds (except the esd in the dihedral angle between two l.s. planes) are estimated using the full covariance matrix. The cell esds are taken into account individually in the estimation of esds in distances, angles and torsion angles; correlations between esds in cell parameters are only used when they are defined by crystal symmetry. An approximate (isotropic) treatment of cell esds is used for estimating esds involving l.s. planes.
Refinement. Refinement of F^2^ against ALL reflections. The weighted R-factor wR and goodness of fit S are based on F^2^, conventional R-factors R are based on F, with F set to zero for negative F^2^. The threshold expression of F^2^ > 2sigma(F^2^) is used only for calculating R-factors(gt) etc. and is not relevant to the choice of reflections for refinement. R-factors based on F^2^ are statistically about twice as large as those based on F, and R- factors based on ALL data will be even larger. H-atoms attached to carbon were placed in calculated positions (C—H = 0.93 - 0.97 Å) while that attached to nitrogen was placed in a location derived from a difference map and its coordinates adjusted to give N—H = 0.89 %A. All were included as riding contributions with isotropic displacement parameters 1.2 - 1.5 times those of the attached atoms.

### Fractional atomic coordinates and isotropic or equivalent isotropic displacement parameters (Å^2^)

**Table:**

	*x*	*y*	*z*	*U*_iso_*/*U*_eq_	
Br1	0.42103 (4)	0.22083 (6)	0.82606 (3)	0.0970 (2)	
O1	0.18452 (12)	0.1239 (2)	0.63926 (13)	0.0510 (4)	
O2	0.48438 (13)	−0.0886 (2)	0.61836 (13)	0.0529 (4)	
N1	0.33335 (14)	−0.0021 (2)	0.65056 (13)	0.0403 (4)	
N2	0.37878 (14)	0.0634 (2)	0.51316 (14)	0.0424 (4)	
H2	0.417288	0.072482	0.468641	0.051*	
C1	0.28014 (15)	0.1311 (3)	0.50632 (15)	0.0361 (4)	
C2	0.25666 (15)	0.0868 (3)	0.60614 (15)	0.0374 (4)	
C3	0.40774 (16)	−0.0157 (3)	0.59400 (16)	0.0395 (5)	
C4	0.3411 (2)	−0.0664 (3)	0.74680 (19)	0.0549 (6)	
H4A	0.349683	−0.177478	0.743311	0.066*	
H4B	0.279528	−0.047609	0.770075	0.066*	
C5	0.4249 (3)	−0.0010 (5)	0.8179 (2)	0.0752 (9)	
H5A	0.487132	−0.031874	0.799497	0.090*	
H5B	0.422518	−0.044378	0.880776	0.090*	
C6	0.20839 (17)	0.0547 (3)	0.42505 (16)	0.0412 (5)	
C7	0.12239 (18)	−0.0173 (3)	0.4386 (2)	0.0513 (6)	
H7	0.104522	−0.017699	0.499314	0.062*	
C8	0.0619 (2)	−0.0895 (4)	0.3625 (3)	0.0659 (8)	
H8	0.003723	−0.137210	0.372572	0.079*	
C9	0.0871 (3)	−0.0910 (4)	0.2741 (3)	0.0803 (10)	
H9	0.046718	−0.140238	0.223419	0.096*	
C10	0.1722 (4)	−0.0197 (6)	0.2595 (3)	0.1016 (15)	
H10	0.189448	−0.020397	0.198523	0.122*	
C11	0.2333 (3)	0.0538 (5)	0.3343 (2)	0.0772 (10)	
H11	0.290963	0.102283	0.323391	0.093*	
C12	0.28154 (17)	0.3076 (3)	0.49925 (17)	0.0406 (5)	
C13	0.3654 (2)	0.3905 (3)	0.5354 (3)	0.0680 (8)	
H13	0.422881	0.338705	0.562487	0.082*	
C14	0.3647 (3)	0.5504 (4)	0.5316 (3)	0.0851 (11)	
H14	0.421780	0.605165	0.556105	0.102*	
C15	0.2814 (3)	0.6282 (4)	0.4925 (3)	0.0778 (10)	
H15	0.281492	0.735614	0.489416	0.093*	
C16	0.1985 (3)	0.5476 (4)	0.4581 (3)	0.0750 (9)	
H16	0.141001	0.600601	0.432518	0.090*	
C17	0.1975 (2)	0.3870 (3)	0.4603 (2)	0.0598 (7)	
H17	0.140013	0.333439	0.435486	0.072*	

### Atomic displacement parameters (Å^2^)

**Table:**

	*U*^11^	*U*^22^	*U*^33^	*U*^12^	*U*^13^	*U*^23^
Br1	0.1041 (4)	0.0862 (3)	0.0912 (3)	−0.0175 (2)	−0.0107 (2)	−0.0181 (2)
O1	0.0413 (9)	0.0627 (11)	0.0519 (9)	0.0039 (8)	0.0162 (7)	−0.0076 (8)
O2	0.0477 (9)	0.0542 (10)	0.0584 (10)	0.0179 (8)	0.0137 (8)	0.0165 (8)
N1	0.0412 (9)	0.0405 (10)	0.0407 (9)	0.0009 (7)	0.0112 (7)	0.0045 (8)
N2	0.0378 (9)	0.0478 (11)	0.0440 (10)	0.0112 (8)	0.0138 (7)	0.0076 (8)
C1	0.0328 (9)	0.0361 (10)	0.0399 (10)	0.0045 (8)	0.0074 (8)	−0.0002 (8)
C2	0.0359 (10)	0.0365 (11)	0.0402 (10)	−0.0016 (8)	0.0076 (8)	−0.0051 (8)
C3	0.0393 (11)	0.0347 (11)	0.0458 (11)	0.0024 (8)	0.0108 (9)	0.0030 (9)
C4	0.0610 (15)	0.0567 (15)	0.0492 (13)	−0.0017 (12)	0.0155 (11)	0.0142 (12)
C5	0.080 (2)	0.088 (3)	0.0543 (16)	0.0110 (17)	0.0019 (15)	0.0095 (16)
C6	0.0456 (11)	0.0348 (11)	0.0421 (11)	0.0037 (9)	0.0038 (9)	−0.0017 (9)
C7	0.0402 (11)	0.0536 (14)	0.0587 (14)	0.0015 (10)	0.0038 (10)	−0.0107 (12)
C8	0.0479 (14)	0.0576 (17)	0.086 (2)	−0.0004 (12)	−0.0069 (13)	−0.0180 (15)
C9	0.089 (2)	0.072 (2)	0.069 (2)	−0.0047 (18)	−0.0169 (17)	−0.0243 (17)
C10	0.127 (3)	0.131 (4)	0.0463 (17)	−0.038 (3)	0.0114 (19)	−0.022 (2)
C11	0.095 (2)	0.092 (2)	0.0469 (15)	−0.032 (2)	0.0203 (15)	−0.0127 (16)
C12	0.0421 (11)	0.0354 (11)	0.0438 (11)	0.0011 (8)	0.0055 (9)	−0.0012 (9)
C13	0.0529 (15)	0.0466 (15)	0.095 (2)	−0.0047 (12)	−0.0158 (14)	0.0038 (14)
C14	0.077 (2)	0.0505 (17)	0.117 (3)	−0.0192 (16)	−0.015 (2)	−0.0067 (18)
C15	0.094 (2)	0.0365 (14)	0.100 (3)	−0.0010 (15)	0.007 (2)	−0.0063 (15)
C16	0.0706 (19)	0.0417 (15)	0.107 (3)	0.0166 (14)	−0.0011 (18)	0.0032 (16)
C17	0.0461 (13)	0.0425 (13)	0.085 (2)	0.0069 (10)	−0.0042 (13)	−0.0032 (13)

### Geometric parameters (Å, º)

**Table:**

Br1—C5	1.923 (4)	C7—H7	0.9300
O1—C2	1.207 (3)	C8—C9	1.351 (5)
O2—C3	1.223 (3)	C8—H8	0.9300
N1—C2	1.366 (3)	C9—C10	1.366 (6)
N1—C3	1.402 (3)	C9—H9	0.9300
N1—C4	1.455 (3)	C10—C11	1.386 (5)
N2—C3	1.333 (3)	C10—H10	0.9300
N2—C1	1.461 (3)	C11—H11	0.9300
N2—H2	0.8899	C12—C17	1.374 (3)
C1—C6	1.529 (3)	C12—C13	1.378 (4)
C1—C12	1.530 (3)	C13—C14	1.384 (5)
C1—C2	1.546 (3)	C13—H13	0.9300
C4—C5	1.502 (5)	C14—C15	1.360 (5)
C4—H4A	0.9700	C14—H14	0.9300
C4—H4B	0.9700	C15—C16	1.352 (5)
C5—H5A	0.9700	C15—H15	0.9300
C5—H5B	0.9700	C16—C17	1.389 (4)
C6—C7	1.375 (4)	C16—H16	0.9300
C6—C11	1.381 (4)	C17—H17	0.9300
C7—C8	1.390 (4)		
			
C2—N1—C3	111.28 (18)	C6—C7—C8	120.6 (3)
C2—N1—C4	125.0 (2)	C6—C7—H7	119.7
C3—N1—C4	123.6 (2)	C8—C7—H7	119.7
C3—N2—C1	113.63 (18)	C9—C8—C7	120.5 (3)
C3—N2—H2	121.6	C9—C8—H8	119.8
C1—N2—H2	124.8	C7—C8—H8	119.8
N2—C1—C6	110.32 (18)	C8—C9—C10	119.5 (3)
N2—C1—C12	112.45 (18)	C8—C9—H9	120.2
C6—C1—C12	113.30 (17)	C10—C9—H9	120.2
N2—C1—C2	99.99 (16)	C9—C10—C11	120.9 (3)
C6—C1—C2	111.75 (18)	C9—C10—H10	119.5
C12—C1—C2	108.26 (17)	C11—C10—H10	119.5
O1—C2—N1	126.1 (2)	C6—C11—C10	119.8 (3)
O1—C2—C1	126.7 (2)	C6—C11—H11	120.1
N1—C2—C1	107.18 (17)	C10—C11—H11	120.1
O2—C3—N2	128.5 (2)	C17—C12—C13	118.6 (2)
O2—C3—N1	123.8 (2)	C17—C12—C1	120.4 (2)
N2—C3—N1	107.71 (18)	C13—C12—C1	120.9 (2)
N1—C4—C5	114.0 (2)	C12—C13—C14	120.4 (3)
N1—C4—H4A	108.8	C12—C13—H13	119.8
C5—C4—H4A	108.8	C14—C13—H13	119.8
N1—C4—H4B	108.8	C15—C14—C13	120.7 (3)
C5—C4—H4B	108.8	C15—C14—H14	119.6
H4A—C4—H4B	107.7	C13—C14—H14	119.6
C4—C5—Br1	113.0 (2)	C16—C15—C14	119.2 (3)
C4—C5—H5A	109.0	C16—C15—H15	120.4
Br1—C5—H5A	109.0	C14—C15—H15	120.4
C4—C5—H5B	109.0	C15—C16—C17	121.2 (3)
Br1—C5—H5B	109.0	C15—C16—H16	119.4
H5A—C5—H5B	107.8	C17—C16—H16	119.4
C7—C6—C11	118.6 (2)	C12—C17—C16	119.9 (3)
C7—C6—C1	123.3 (2)	C12—C17—H17	120.1
C11—C6—C1	118.0 (2)	C16—C17—H17	120.1
			
C3—N2—C1—C6	−113.5 (2)	N2—C1—C6—C11	−54.9 (3)
C3—N2—C1—C12	119.0 (2)	C12—C1—C6—C11	72.1 (3)
C3—N2—C1—C2	4.3 (2)	C2—C1—C6—C11	−165.2 (3)
C3—N1—C2—O1	−176.7 (2)	C11—C6—C7—C8	0.0 (4)
C4—N1—C2—O1	−0.6 (4)	C1—C6—C7—C8	−177.7 (2)
C3—N1—C2—C1	3.1 (2)	C6—C7—C8—C9	0.4 (5)
C4—N1—C2—C1	179.1 (2)	C7—C8—C9—C10	−0.6 (6)
N2—C1—C2—O1	175.5 (2)	C8—C9—C10—C11	0.2 (7)
C6—C1—C2—O1	−67.8 (3)	C7—C6—C11—C10	−0.4 (6)
C12—C1—C2—O1	57.7 (3)	C1—C6—C11—C10	177.4 (4)
N2—C1—C2—N1	−4.3 (2)	C9—C10—C11—C6	0.3 (7)
C6—C1—C2—N1	112.5 (2)	N2—C1—C12—C17	157.7 (2)
C12—C1—C2—N1	−122.08 (19)	C6—C1—C12—C17	31.7 (3)
C1—N2—C3—O2	177.4 (2)	C2—C1—C12—C17	−92.8 (3)
C1—N2—C3—N1	−2.8 (3)	N2—C1—C12—C13	−25.2 (3)
C2—N1—C3—O2	179.4 (2)	C6—C1—C12—C13	−151.1 (3)
C4—N1—C3—O2	3.3 (4)	C2—C1—C12—C13	84.3 (3)
C2—N1—C3—N2	−0.4 (3)	C17—C12—C13—C14	−0.5 (5)
C4—N1—C3—N2	−176.5 (2)	C1—C12—C13—C14	−177.7 (3)
C2—N1—C4—C5	−113.9 (3)	C12—C13—C14—C15	0.1 (7)
C3—N1—C4—C5	61.7 (4)	C13—C14—C15—C16	0.8 (7)
N1—C4—C5—Br1	55.0 (3)	C14—C15—C16—C17	−1.3 (7)
N2—C1—C6—C7	122.8 (2)	C13—C12—C17—C16	0.0 (5)
C12—C1—C6—C7	−110.1 (3)	C1—C12—C17—C16	177.2 (3)
C2—C1—C6—C7	12.5 (3)	C15—C16—C17—C12	0.9 (6)

### Hydrogen-bond geometry (Å, º)

*Cg*3 is the centroid of the C12–C17 benzene ring.

**Table:**

*D*—H···*A*	*D*—H	H···*A*	*D*···*A*	*D*—H···*A*
N2—H2···O2^i^	0.89	1.98	2.862 (3)	174
C4—H4*A*···O1^ii^	0.97	2.49	3.175 (3)	128
C8—H8···O1^iii^	0.93	2.56	3.387 (3)	148
C10—H10···*Cg*3^iv^	0.93	2.85	3.771 (5)	173

Symmetry codes: (i) −*x*+1, −*y*, −*z*+1; (ii) −*x*+1/2, *y*−1/2, −*z*+3/2; (iii) −*x*, −*y*, −*z*+1; (iv) −*x*+1/2, *y*−1/2, −*z*+1/2.
